# Progress towards non-invasive diagnosis and follow-up of celiac disease in children; a prospective multicentre study to the usefulness of plasma I-FABP

**DOI:** 10.1038/s41598-017-07242-4

**Published:** 2017-08-17

**Authors:** M. P. M. Adriaanse, A. Mubarak, R. G. Riedl, F. J. W. Ten Kate, J. G. M. C. Damoiseaux, W. A. Buurman, R. H. J. Houwen, A. C. E. Vreugdenhil, Celiac Disease Study Group, M. C. G. Beeren, C. M. L. van Dael, A. C. Engelberts, J. H. Hanekom, J. J. E. Hendriks, T. Hubregtse, R. Jonkers, L. S. Kapteijns, E. M. Kerkvliet, A. M. van den Neucker, S. Potgieter, J. Raaijmakers, P. P. R. Rosias, P. J. H. M. Stouthart, P. M. V. M. Theunissen, H. M. van Wering, J. B. G. Wijnands

**Affiliations:** 1 Department of Pediatrics & Nutrition and Toxicology Research Institute Maastricht (NUTRIM), Maastricht University Medical Centre, Maastricht, The Netherlands; 2 Department of Pediatric Gastroenterology, Wilhelmina Children’s Hospital, Utrecht, The Netherlands; 3 Department of Pathology, Maastricht University Medical Centre, Maastricht, The Netherlands; 4 Department of Pathology, Wilhelmina Children’s Hospital, Utrecht, The Netherlands; 5 Central Diagnostic Laboratory, Maastricht University Medical Centre, Maastricht, The Netherlands; 6 Department of General Surgery, Maastricht University Medical Centre, Maastricht, The Netherlands; 7 Department of Neuroscience, School of Mental Health and Neuroscience, Maastricht University Medical Centre, Maastricht, The Netherlands; 8 Department of Pediatrics, Sint Anna Hospital, Geldrop, The Netherlands; 9 Department of Pediatrics, VieCuri, Venlo The Netherlands; 10 Department of Pediatrics, Zuyderland, Sittard The Netherlands; 11 Department of Pediatrics, Laurentius Hospital, Roermond, The Netherlands; 12 Department of Pediatrics, Sint Jans Gasthuis, Weert, The Netherlands; 13 Department of Pediatrics, Amphia Hospital Breda, Weert, The Netherlands; 14 Department of Pediatrics, Zuyderland, Heerlen The Netherlands

## Abstract

This prospective study investigates whether measurement of plasma intestinal-fatty acid binding protein (I-FABP), a sensitive marker for small intestinal epithelial damage, improves non-invasive diagnosing of celiac disease (CD), and whether I-FABP levels are useful to evaluate mucosal healing in patients on a gluten-free diet (GFD). Ninety children with elevated tTG-IgA titres and HLA-DQ2/DQ8 positivity were included (study group). Duodenal biopsies were taken, except in those fulfilling the ESPGHAN criteria. Plasma I-FABP levels and tTG-IgA titres were assessed sequentially during six months of follow-up. Eighty children with normal tTG-IgA titres served as control group. In 61/90 (67.8%) of the children in the study group an increased I-FABP level was found; in all these children CD diagnosis was confirmed. Interestingly, in 14/30 (46.7%) children with slightly elevated tTG-IgA titres (<10x upper limit of normal), an increased I-FABP level was found. In all these children the diagnosis of CD was confirmed histologically. After gluten elimination for six weeks I-FABP levels had decreased towards levels in the control group. Measurement of plasma I-FABP, in addition to tTG-IgA, EMA-IgA and HLAtyping, enables non-invasive diagnosing of CD in a substantial number of children, and might therefore be of value in the diagnostic approach of CD.

## Introduction

Celiac disease (CD) is a common immune-mediated enteropathy characterized by gluten-induced small intestinal damage with loss of absorptive villi in genetically susceptible individuals^1^. Traditionally, the diagnosis of CD relies on objectifying the typical small intestinal lesions by a duodenal biopsy. While progress has been made, the absence of non-invasive methods for evaluation of intestinal damage and villous atrophy, both at diagnosis and during follow-up of patients on a gluten-free diet (GFD), remains a target for improvement in the clinical management of CD.

Introduction of the revised guidelines for CD by the European Society for Paediatric Gastroenterology, Hepatology and Nutrition (ESPGHAN) in 2012 has permitted a non-invasive diagnosis in selected patients^2^. In summary, a duodenal biopsy can be avoided in children with a clinical picture of CD, human leukocyte antigen (HLA) genotype DQ2 and/or DQ8 and strongly elevated anti-tissue transglutaminase autoantibody (tTG-IgA) titres, verified by anti-endomysium autoantibody (EMA-IgA) positivity. However, in patients not fulfilling all criteria the final diagnosis relies on duodenal biopsy, which is associated with high costs, inconvenience for the child and its parents, while interpretation difficulties due to patchy lesions and inadequate biopsy specimen may occur^3–6^.

Apart from these diagnostic concerns, a reliable marker to evaluate CD activity in patients on a GFD is needed. Although CD autoantibodies are suggested to quantify disease activity at time of presentation, various studies demonstrate that autoantibody titres are unreliable markers of intestinal healing^7–10^, making these imperfect tools for this goal.

Intestinal-fatty acid binding protein (I-FABP) has emerged as a valuable marker in the evaluation of intestinal epithelial damage in various diseases such as mesenteric infarction, necrotising enterocolitis and intestinal ischemia^11–14^. I-FABP is a small cytosolic protein (15 kDa) present in mature enterocytes that is rapidly released into the systemic circulation upon enterocyte damage^11, 15^. Therefore, circulating I-FABP may provide actual information about the extent of intestinal epithelial cell injury. Our retrospective studies indeed showed elevated levels of I-FABP in children and adults with untreated CD, and rapid recovery and normalization of these levels after initiation of a GFD^16–18^. The results of these studies suggest that elevated I-FABP levels in children with elevated CD autoantibody titres and an HLA-DQ2 and/or -DQ8 genotype confirm a diagnosis of CD, making a biopsy unnecessary. However, the retrospective study design hampered decision-making based on I-FABP level in these children, and hindered standardized follow-up of I-FABP levels after initiation of a GFD.

The aim of the present study is to prospectively investigate the value of plasma I-FABP level in the current diagnostic algorithm for CD in children, and to evaluate whether I-FABP level in addition to tTG-IgA titre and HLA genotyping improves non-invasive diagnosing of CD. Moreover, this study investigated the usefulness of plasma I-FABP to monitor disease activity in children on a GFD.

## Methods

### Subjects

Children (6 months–18 years) presenting with a clinical suspicion of CD, elevated CD autoantibody titres, and a HLA-DQ2 and/or -DQ8 genotype at the Maastricht University Medical Centre (MUMC), Wilhelmina Children’s Hospital Utrecht (WKZ), and their affiliated hospitals, the Netherlands, between March 2010 and August 2013, were prospectively included in this study. Clinical suspicion of CD was defined as the presence of (extra-) intestinal symptoms suggestive for CD and/or a high risk for CD, either being a positive family history or suffering from an associated autoimmune disease (type I diabetes mellitus, auto-immune hepatitis, autoimmune thyroid disease), Down syndrome, Turner syndrome, Williams syndrome, or cystic fibrosis. Children with a history of inflammatory bowel disease were excluded from this study, since this condition in itself might result in increased I-FABP levels^19^. Data from children who completely finished the study protocol (referred to as the study group) were used for analysis.

The control group consisted of 80 children presenting at the outpatient clinic of the MUMC with a clinical suspicion of CD, yet with normal tTG-IgA and IgA-EMA titres.

The study was conducted according to the Declaration of Helsinki. The study received IRB approval of the Medical Ethical Committee (METC) MUMC and the METC University Medical Centre Utrecht. Written informed consent was obtained from the parents, and of children older than 11 years.

### Study protocol

At time of presentation CD autoantibody titres, HLA genotype and plasma I-FABP level were determined in all included children. In children with elevated CD autoantibody titres and HLA-DQ2 and/or -DQ8 genotype, duodenal biopsies were obtained from the descending duodenum and duodenal bulb. Histological evaluation was not performed in patients presenting after the introduction of the revised ESPGHAN guideline for CD in January 2012 and who fulfilled all four criteria for a diagnosis without biopsy (tTG-IgA level >10 x upper normal limit (>100 U/ml)) accompanied by symptoms, HLA-DQ2 and/or -DQ8 genotype and EMA-IgA positivity)^2^.

**Figure 1:**
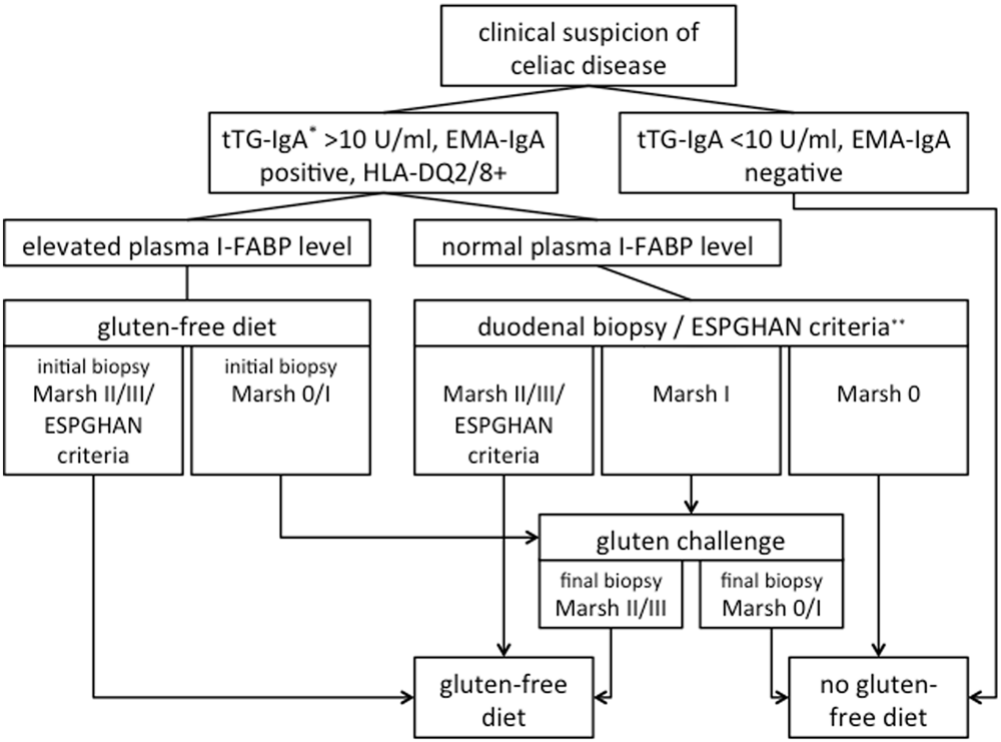
Study protocol.

In order to test our hypothesis that elevated I-FABP levels are highly predictive of CD in children with a tTG-IgA titre >10 U/ml, all these children started a GFD if the plasma I-FABP level was above the cut-off value of 450 pg/ml, independent of confirmation of the disease, either through the new ESPGHAN guidelines or the biopsy result, and with parental consent (Fig. 1).

As our former retrospective study indicates that a normal I-FABP level does not exclude a diagnosis of CD^17^, the diagnostic procedure in children of the study group with elevated CD autoantibody titres, HLA-DQ2 and/or -DQ8 genotype and a normal I-FABP level, complied with the ESPGHAN guidelines^2, 20^; i.e. the diagnosis of CD was confirmed in patients with Marsh grade II or III at duodenal biopsy, and in patients fulfilling all ESPGHAN 2012 criteria for a non-invasive diagnosis, as mentioned above. These patients started a GFD as well. In patients presenting with a tTG-IgA > 10 U/ml, a normal I-FABP level and a normal duodenal biopsy (Marsh 0), CD diagnosis was rejected and patients continued on a normal gluten-containing diet. A final diagnosis could not be established in patients presenting with a normal I-FABP level and Marsh grade I. Therefore, these children started a six-month GFD followed by a three-month gluten challenge with 375 mg/kg/day dietary gluten to reassess the diagnosis in agreement with the guidelines^2, 20^. This period of gluten intake was both preceded and followed by a duodenal biopsy to evaluate the histological response to dietary gluten intake. This gluten challenge procedure was also performed to reassess the diagnosis in children with an elevated I-FABP level and a Marsh grade 0 or I at the initial duodenal biopsy.

At the end of the study, a final diagnosis of CD was established in patients with elevated CD autoantibody titres and HLA-DQ2 and/or -DQ8 genotype who showed crypt hyperplasia (Marsh II) and/or villous atrophy (Marsh III) at initial or final duodenal biopsy, and in patients fulfilling all criteria for a non-invasive diagnosis according to the ESPGHAN guideline 2012^2^. The diagnosis of CD was rejected when the duodenal biopsy after gluten challenge showed Marsh 0 or I. Violation of the study protocol or withdrawal from the study by children for any reason was defined as a reason for exclusion from the study.

### Study visits during follow-up

**Figure 2:**
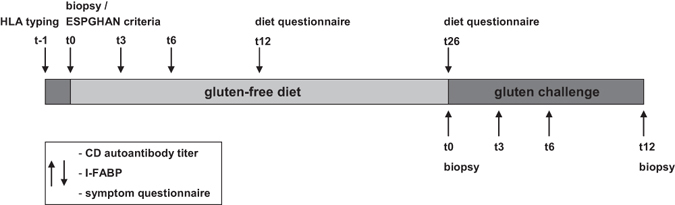
Study visits and measurements at disease presentation and during follow-up of all included patients.

Patients who were started on a GFD visited the outpatient clinic after 3, 6, 12 and 26 weeks upon gluten elimination (Fig. 2). An experienced dietician informed the child and its parents and guided them during the follow-up. The dietician or paediatrician evaluated dietary adherence at 3 and 6 months after initiation of therapy using a standard questionnaire (scoring for dietary strictness, unintended gluten intake and environmental factors). Patients who started a gluten challenge visited the outpatient clinic at 0, 3, 6 and 12 weeks of gluten intake. Patients with both a normal I-FABP level and a normal duodenal biopsy who continued the gluten-containing diet visited the outpatient clinic at 3 and 6 months after initial presentation.

During the study visits, a symptom questionnaire (scoring for abdominal pain, nausea, diarrhoea and constipation, appetite, fatigue, irritability and psychological complaints) was conducted by the paediatrician (Fig. 2).

### Sample collection

Blood samples for CD autoantibody, HLA genotyping and I-FABP measurement from all included children were obtained at the time of first presentation with a clinical suspicion of CD or during the endoscopy for the provision of duodenal biopsies. Furthermore, blood sampling for CD autoantibody and I-FABP measurement was performed during every study visit (Fig. 2).

### Celiac disease autoantibody measurement

Blood samples for tTG-IgA and EMA-IgA titre measurement were handled for 12 minutes by 3000 rpm at 20 °C to derive serum and were analysed in routine laboratory practice. Serum EMA-IgA titre was determined by means of commercial indirect immunofluorescence tests (Scimedx, Densvill, NJ or Euroimmun, Luebeck, Germany). Serum tTG-IgA titre was analysed using a quantitative automated fluorescent-enzyme immuno-assay (FEIA, Thermo Fisher Scientific, Freiburg, Germany, upper limit of normal 10 U/ml). Strongly elevated tTG-IgA titres were defined as >10x the upper limit of normal (>100 U/ml). Total serum IgA titre was measured to evaluate IgA-deficiency (total IgA < 0.07 g/l) using nephelometry (Beckman Coulter, Mijdrecht, the Netherlands). In IgA-deficient patients (n = 3), tTG-IgG titres were determined using a quantitative automated FEIA (Thermo Fisher Scientific).

### Human leukocyte antigen typing

DNA was extracted using Qia-AMP kits (Qiagen, Hilden, Germany). HLA-DRB1, -DQB1 and -DQA1 typing was performed by Luminex SSO^21^. If DQB1*03 was present high resolution typing was performed using sequencing-based typing^22^. This method enabled identification of HLA-DQ2 (DQA1*05-DQB1*02 (DQ2.5) and DQA1*02-DQB1*02 (DQ2.2)) and HLA-DQ8 (DQA1*03-DQB1*03:02).

### Intestinal-fatty acid binding protein measurement

Blood samples for I-FABP analysis were handled for 15 minutes by 3500 rpm at 4 °C to derive plasma. Samples from time of presentation were analysed directly to use the result for decision-making. Follow-up samples were stored at −80 °C until batch analysis. Plasma I-FABP levels were determined in duplicate using a highly specific in-house enzyme-linked immunosorbent assay (ELISA) that selectively detects human I-FABP (standard: 12.5–800 pg/ml) developed at the Department of General Surgery of the MUMC. Storage time of the plasma samples did not significantly influence the levels of I-FABP (unpublished data). The capture and detection antibodies used for the I-FABP ELISA were different from those used in our previous study. To calculate the optimal cut-off point to discriminate villous atrophy from normal intestinal mucosa, a receiver operating characteristic (ROC) curve was performed prior to this study on samples of CD (n = 38) and control children (n = 40) in stock, revealing an optimal cut-off point of 450 pg/ml with a positive predictive value of 87.5% (sensitivity of 92.1%, specificity 87.7%).

### Histology

Endoscopy was performed according to the ESPGHAN guidelines. At least four biopsies were taken from the descending duodenum and at least two from the duodenal bulb. Two experienced pathologists assessed the histological slides independently (R.R. and F.T.K.), and were blinded for clinical information. Mucosal damage was graded according to the modified Marsh classification: Marsh 0 normal biopsy, Marsh I intraepithelial lymphocytosis (IEL), Marsh II crypt hyperplasia, Marsh IIIA partial villous atrophy, Marsh IIIB subtotal villous atrophy and Marsh IIIC total villous atrophy^23, 24^. Additional CD3-staining was performed whenever there was doubt about the number of intraepithelial lymphocytes. The most severe grade of damage observed was reported. In patients with discrepancy in Marsh grade between the two pathologists, both pathologists reassessed the histological slides, in case of ongoing discrepancy despite reassessment, patients were excluded from the study. Endoscopy with duodenal biopsy was not performed in the control group.

### Statistical analysis

Statistical analyses were performed using SPSS, version 20.0 for Windows (SPSS Inc, Chicago, IL). Normality was tested using the Kolmogorov-Smirnov test. For descriptive purposes, plasma I-FABP levels (right-skewed distribution) are presented as median and interquartile range. A Mann-Whitney U test was used for between-group comparisons for continuous data. Log transformed (ln) baseline I-FABP levels were compared between CD patients and controls with a linear regression model adjusting for age. CD patients were stratified for Marsh grade, and compared for plasma I-FABP levels using the Kruskal Wallis test. Spearman’s correlation coefficient test was used for identifying a correlation between plasma I-FABP levels, tTG-IgA titres (IgA-deficient patients were excluded for this analysis) and age. A p-value below 0.05 was considered statistically significant.

## Results

### Baseline characteristics

One hundred eight children with a clinical suspicion of CD, elevated CD autoantibody titres, and HLA-DQ2 and/or -DQ8 were enrolled. Eighteen children did not comply with the study protocol and were excluded from the data analysis. In this group, nine patients with proven CD (diagnosed either by duodenal biopsy or ESPGHAN criteria for a non-invasive diagnosis) were withdrawn because of rejection of blood drawing during follow-up. Four children were excluded because of discrepancy in biopsy assessment between both pathologists, affecting the final diagnosis. Five children with Marsh I were excluded since their parents did not give consent for a complete gluten challenge. While three of these children had a normal plasma I-FABP level, two showed an elevated plasma I-FABP level at time of presentation. The first child, suffering from type I diabetes mellitus, was asymptomatic and presented with a slightly elevated tTG-IgA titre (25 U/ml), EMA-IgA antibodies, HLA-DQ2.5 and -DQ8 positivity, and Marsh I at duodenal biopsy. Based on these results and the elevated I-FABP level, a GFD was started, as per protocol, resulting in normalization of both CD autoantibody titres and plasma I-FABP level within six months. A three-month gluten challenge was started after one year of gluten elimination, resulting in a tTG-IgA titre of 82 U/ml, re-emergence of EMA-IgA antibodies, and an elevated I-FABP level. Parents did not agree on a second duodenal biopsy to make a final diagnosis. The other child presented with a slightly elevated tTG-IgA titre (11 U/ml), EMA-IgA antibodies, HLA-DQ2.5 positivity, Marsh grade I at duodenal biopsy and an elevated I-FABP level. However, parents did not agree on gluten elimination and the patient continued the normal gluten-containing diet. Plasma I-FABP level and tTG-IgA titre were monitored at three and six months after presentation. None of the levels returned to normal, with tTG-IgA varying from 11–16 U/ml and I-FABP varying from 387–522 pg/ml. The remaining 90 patients are further mentioned as the study group. Baseline characteristics of the study group are presented in Table 1.

**Table 1:** Baseline characteristics of the study group.

	Study group n = 90
Mean age (years)	7.3 years (±4.4)
Female/male	58 (64.4%)/32 (35.6%)
*tTG titre*	
• >100 U/ml	60 (66.7%)
• 10–100 U/ml/tTG-IgG positive*	30 (33.3%)
*Duodenal biopsy at time of presentation*	
• Marsh 0	8 (8.9%)
• Marsh I	3 (3.3%)
• Marsh II	0 (0%)
• Marsh IIIA	15 (16.7%)
• Marsh IIIB	26 (28.9%)
• Marsh IIIC	17 (18.9%)
• Not performed**	21 (23.3%)

^*^tTG-IgG titres were measured in three patients because of IgA-deficiency. **Celiac disease diagnosis was established without duodenal biopsy based on the ESPGHAN guideline.

Eighty children with a clinical suspicion of CD, but with normal CD autoantibody titres were included in the control group. No significant difference was present in age between both groups (mean age in control group 8.1 years (±5.5 years), p = 0.130). However, females were overrepresented in the study group as 37 females (52.1%) were present in the control group.

### Plasma I-FABP levels at presentation

**Figure 3:**
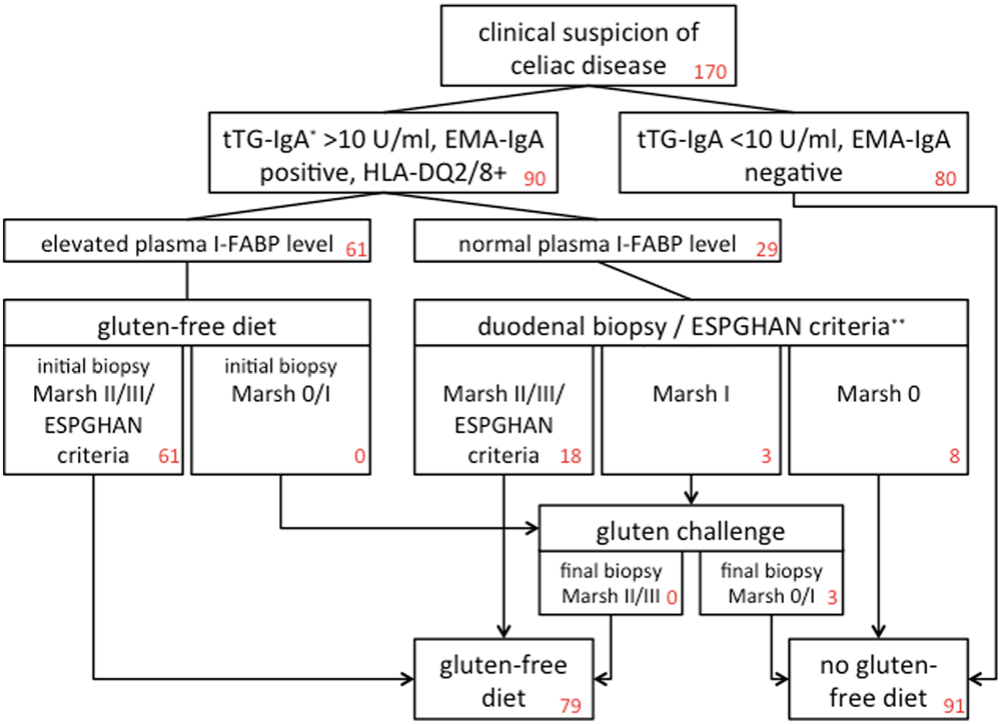
Plasma I-FABP results of all included patients with a clinical suspicion of celiac disease.

At time of presentation, 61/90 (67.8%) patients with a tTG-IgA > 10 U/ml showed a plasma I-FABP level above the cut-off value of 450 pg/ml and started a GFD (Fig. 3). All these patients were directly confirmed as having CD, either by the finding of villous atrophy and/or crypt hyperplasia (Marsh II or Marsh III) at duodenal biopsy (n = 47) or by fulfilling the revised ESPGHAN guideline criteria (n = 14) at the time of presentation.

Twenty-nine (32.2%) patients with a tTG-IgA > 10 U/ml showed an I-FABP level below the cut-off value at presentation, and a final diagnosis was made according to the ESPGHAN guideline. A diagnosis of CD was established in 18 patients; 11 patients showed Marsh grade II (n = 0) or Marsh grade III (Marsh IIIA in 3 patients, Marsh IIIB in 5 patients and Marsh IIIC in 3 patients), while in 7 patients the diagnosis could be confirmed based on the quartet of typical symptoms, a strongly elevated tTG-IgA titre (>100 U/ml), positive EMA-tTG, and HLA-DQ2 and/or -DQ8^2^. These patients started a GFD. Eleven patients with an I-FABP level below the cut-off value did not fulfil the criteria for CD at time of presentation and a CD diagnosis was rejected (n = 8) or reassessed by a gluten challenge procedure (n = 3, none of these patients showed Marsh II or III after gluten challenge). Thus, 18/79 (22.8%) patients with confirmed CD presented with an I-FABP level below the cut-off value. In 61/79 (77.2%) patients with proven CD, I-FABP levels were above the cut-off value and therefore truly positive (Fig. 3). Consequently, the positive predictive value for CD of an increased I-FABP level in children with elevated CD autoantibody titres and HLA-DQ2 and/or -DQ8 was 100%. The negative predictive value of I-FABP in this group was 50%. Sensitivity and specificity of plasma I-FABP for the detection of celiac disease in these patients were 84.7% and 100%, respectively.

In the control group, 7 (8.8%) children showed a plasma I-FABP level above the cut-off value. All these patients had normal CD autoantibody titres. These patients were diagnosed as having a wheat allergy (n = 1), chronic (functional) diarrhoea (n = 3), or were tested for CD because of type I diabetes mellitus (n = 2) or a family history of CD (n = 1).

Furthermore, we evaluated the usefulness of I-FABP in the subgroup of patients presenting with either a slightly elevated tTG-IgA (<10x upper limit of normal; 10-100 U/ml) or with elevated tTG-IgA titres (in IgA-deficient patients), currently needing a diagnostic duodenal biopsy. A slightly elevated tTG-IgA titre was present in 30 (33.3%) children in the study group. Twenty-one of them had proven CD. Fourteen (46.7%) of these patients showed an I-FABP level above the cut-off value; all were directly diagnosed with CD at time of presentation. This suggests that a CD diagnosis could be final in patients with a slightly elevated tTG-IgA and an elevated plasma I-FABP.

### Plasma I-FABP during follow-up in CD patients upon a gluten-free diet

**Figure 4:**
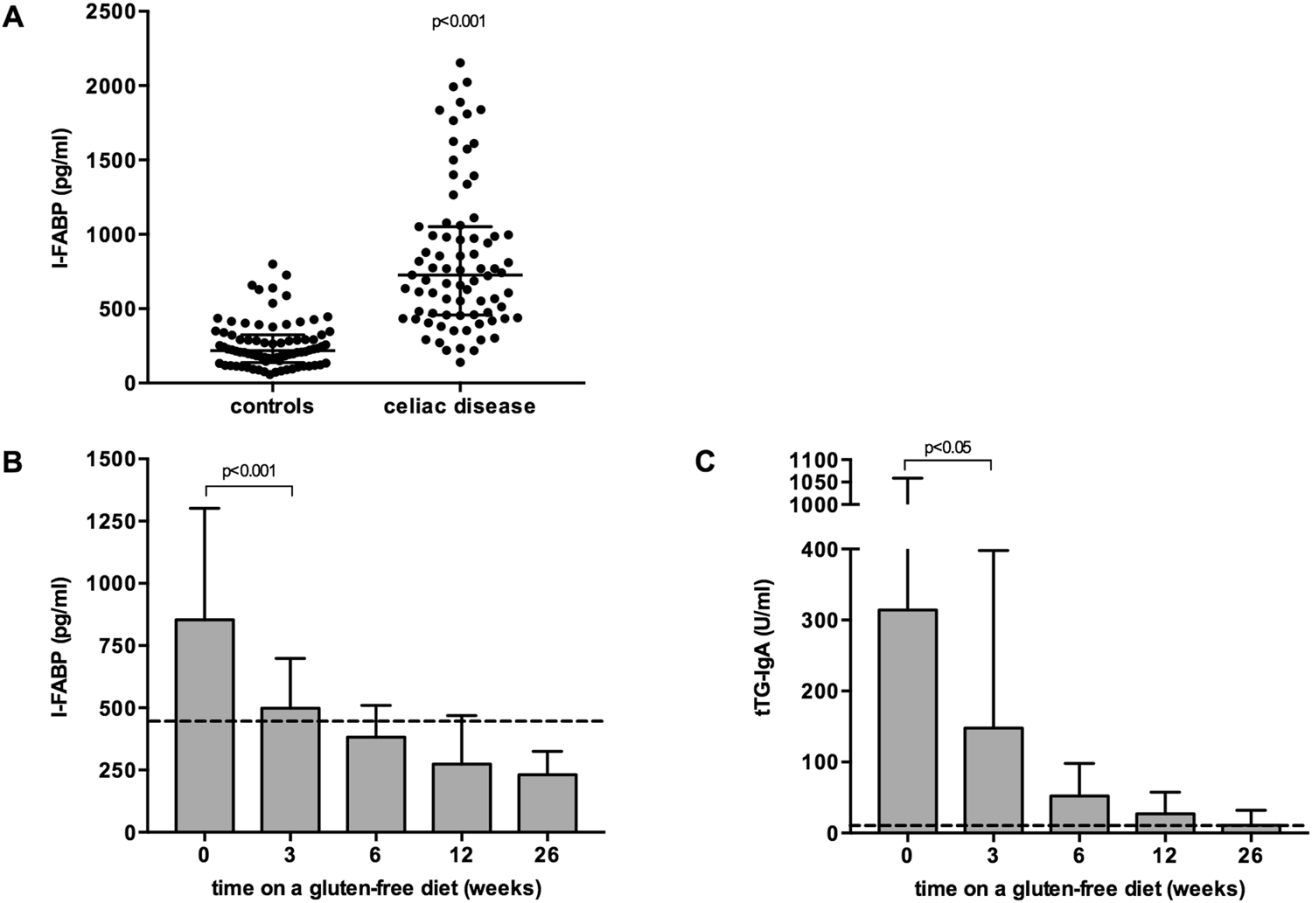
Plasma I-FABP levels are significantly elevated in celiac disease patients compared to control individuals (**A**). Plasma I-FABP levels (**B**) and tTG-IgA titres (**C**) in celiac disease patients after initiation of a gluten-free diet (Figure B includes patients with elevated I-FABP levels at diagnosis only, n = 79, 21, 74, 70, 76 at t = 0, 3, 6, 12 and 26, respectively). *Significantly different as compared with week 0, p < 0.05.

Plasma I-FABP levels were measured sequentially during the first six months of gluten elimination. At presentation, median I-FABP levels in children with confirmed CD were significantly increased as compared with levels in the control group (726 pg/ml [458–1024] versus 218 pg/ml [143–323], p < 0.001) (Fig. 4A). Three weeks of gluten elimination resulted in a significant decrease in plasma I-FABP levels in patients with initially elevated levels (498 pg/ml [270–697] versus 854 pg/ml [635-1265], p < 0.05, Fig. 4B). After 26 weeks of gluten elimination I-FABP levels (231 pg/ml [185–318]) were not significantly different from values found in the control group. Plasma I-FABP in patients with an initially elevated level decreased below the cut-off value in 42.1%, 68.4% (382 pg/ml [253–497]), 70.4% (274 pg/ml [207–469]) and 82.4% (231 pg/ml [185–318]) after 3, 6, 12 and 26 weeks, respectively. Interestingly, I-FABP levels also decreased upon gluten elimination in 16/18 (88.9%) CD patients with an I-FABP level below the cut-off value at diagnosis (data not shown).

Similarly, titres of tTG-IgA decreased in all patients starting a GFD and a significant decrease in tTG-IgA titres was found after three weeks on a GFD (314 U/ml [97–1025] at diagnosis to 148 U/mL [68–363] after 3 weeks, p < 0.05). However, tTG-IgA titres did not normalize within six months of gluten elimination (Fig. 4C). Median tTG-IgA titres after 6, 12 and 26 weeks of gluten elimination were 52 U/ml [18–98], 27 U/ml [9–55], and 12 U/ml [4–31], respectively. tTG-IgA titres normalized in only 4.8%, 8.3%, 27.9% and 47.3% of CD patients after 3, 6, 12 and 26 weeks, respectively.

### Correlation of plasma I-FABP level with histological and serological disease markers at time of disease presentation and during gluten elimination in CD patients

**Figure 5:**
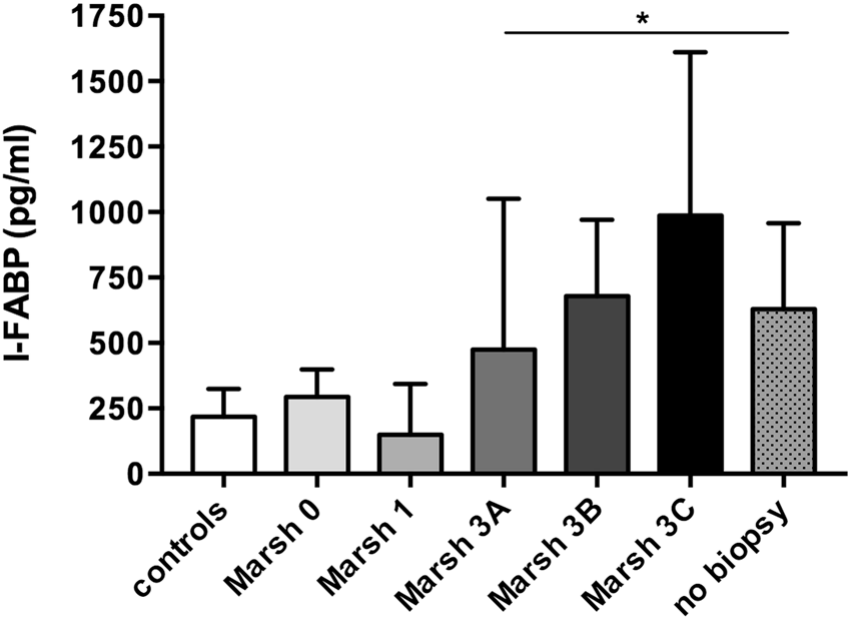
Plasma I-FABP levels stratified for the degree of villous atrophy at time of diagnosis of CD. *Significantly elevated compared with controls, ^#^Significantly elevated compared with Marsh 0, and Marsh I.

Plasma I-FABP level at diagnosis was stratified for the severity of villous atrophy (Fig. 5). Children presenting with Marsh grade IIIC (775 pg/ml [482–1265], p < 0.001), Marsh IIIB (876 pg/ml [583–1070], p < 0.001) and Marsh IIIA (567 pg/ml [466–849], p < 0.001] were found to have significantly elevated I-FABP levels compared to control individuals. Interestingly, also study group patients showing Marsh grade 0 in the initial duodenal biopsy had elevated levels of plasma I-FABP compared with controls.

**Figure 6:**
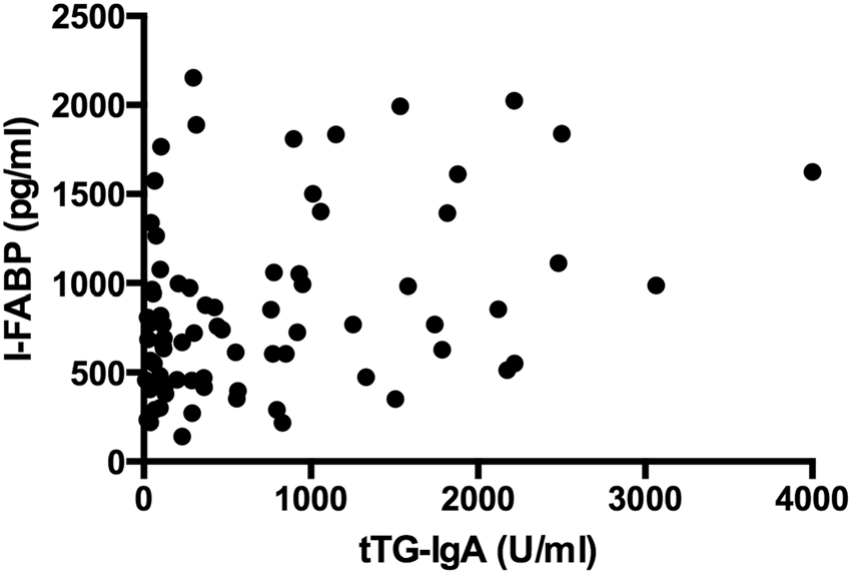
Correlation between plasma I-FABP levels and serum tTG-IgA titres at time of presentation.

At presentation, I-FABP level correlated significantly with tTG-IgA titres (r = 0.346, p < 0.01, Fig. 6), while no correlation was present during follow-up. Plasma I-FABP levels did not correlate with symptoms at presentation or during gluten elimination, nor with dietary adherence when on a GFD (p = 0.511).

## Discussion

The aim of this study was to prospectively investigate whether measurement of plasma I-FABP level, in addition to the currently used parameters, improves the diagnostic algorithm of CD and the monitoring of disease activity in children on a GFD. The data show that use of plasma I-FABP level might improve non-invasive diagnosing in children with elevated CD autoantibody titres and HLA-DQ2 and/or -DQ8 positivity. Furthermore, plasma I-FABP levels decrease rapidly in paediatric CD patients starting a GFD. Our results indicate that plasma I-FABP is a useful additional tool besides the currently used CD autoantibodies and genotyping for evaluation of disease activity at time of diagnosis, and may be useful for monitoring disease activity during follow-up.

A shortcoming in the current diagnostic procedure for CD is the need for invasive duodenal biopsies to confirm the disease in a substantial part of patients. Although the diagnostic accuracy of CD autoantibodies is excellent in well-selected cases and tTG-IgA is even suggested to be a quantitative parameter for disease activity, these markers cannot replace the diagnostic duodenal biopsy in all cases. Endoscopy with duodenal biopsy is an invasive and expensive procedure, and may provide inconclusive or even false results, due to patchy disease, technical limitations and inter-observer variability, especially if severe villous atrophy is missing^3–5, 25^. Appropriate use of a less invasive and more objective tool would add reliability to the diagnosis. Our previous studies have identified I-FABP as a sensitive marker for small intestinal mucosal damage in CD^16–18^. However, these studies have been limited to retrospective data. The prospective nature of the present study allowed decision-making based on I-FABP level and therefore enabled the evaluation of the diagnostic value of plasma I-FABP in children with elevated CD autoantibody titres.

This study demonstrates that all patients with a tTG-IgA above 10 U/ml, HLA-DQ2 and/or -DQ8, and an elevated I-FABP level suffered from CD. According to the revised ESPGHAN guideline, a biopsy remains mandatory in patients with slightly elevated tTG-IgA levels, between 10–100 U/ml. Here we show that out of 30 patients with a tTG-IgA between 10–100 U/ml, the 14 patients with an I-FABP level above 450 pg/ml had histological proven CD. Although this finding requires confirmation, it suggests that a larger group of patients can benefit from a biopsy-free diagnosis of CD than allowed by the current guideline. This would reduce costs, complications and emotional impact on children and their parents.

Furthermore, this study showed rapidly decreasing plasma I-FABP levels in CD children starting a GFD. This quick response to gluten elimination is useful to add reliability to a diagnosis of CD without a duodenal biopsy. In contrast, the tTG-IgA response in CD is delayed in comparison with histological changes^26^. This may be due to its long half-life, or can relate to the fact that CD autoantibodies reflect the immune response rather than mucosal damage. Since follow-up biopsies were not performed in this study, further research is necessary to evaluate whether plasma I-FABP is indeed useful for monitoring mucosal recovery after initiation of treatment, as our results suggest.

Based on the data of this study, we propose to initiate a GFD in all patients with the combination of increased CD autoantibody titres, HLA-DQ2 and -DQ8 and elevated plasma I-FABP level. CD diagnosis can subsequently be considered to be final if I-FABP level, tTG-IgA titre and symptoms respond to a GFD, the mainstay of treatment for CD. By applying this strategy, the number of diagnostic duodenal biopsies needed could be reduced significantly, whereas no patients will be misdiagnosed as having CD. Obviously, in patients with an elevated tTG-IgA titre but an I-FABP level below the cut-off value, and in patients with normal autoantibody titres but a strong clinical suspicion of CD, a duodenal biopsy remains necessary to confirm or reject a diagnosis of CD.

A few limitations apply to this study. While the main strength of this study is the suggestion that a biopsy-free diagnosis is also possible in almost half (14/30) of the children with tTG-IgA between 10–100 U/ml, this should be confirmed in a larger cohort study as the sample size of such patients is limited. Furthermore, due to the invasive nature of the endoscopy follow-up biopsies were not performed. Strict evaluation of the correlation between duodenal histology, I-FABP levels and CD autoantibody titres during gluten elimination was therefore not possible.

In conclusion, this study suggests a new diagnostic approach for patients with suspected CD. Finding an elevated plasma I-FABP level, increased CD autoantibody titres and HLA-DQ2 and/or -DQ8 might be sufficient for the diagnosis of CD and could increase the number of patients that can benefit from a non-invasive approach. Moreover, I-FABP levels recover rapidly after gluten elimination, implying that plasma I-FABP may also be used for monitoring disease activity in CD patients on a GFD.

### Study sponsor

ZonMw: Doelmatigheidsonderzoek. Project number 171001006. The study sponsor did not have a role in the study design, collection, analysis or interpretation of data.
